# Hospitalization expenses for radical prostatectomy in a tertiary hospital in China: a quantile regression and CHAID decision tree analysis

**DOI:** 10.1186/s12913-026-14667-z

**Published:** 2026-05-07

**Authors:** Feiyue Su, Hualian Pei, Yichao Hu, Yiping Li, Weihong Wang, Qinhong Xu

**Affiliations:** 1 Department of Nursing, The First Affiliated Hospital of Ningbo University, Ningbo, 315010 China; 2 Department of Urology, The First Affiliated Hospital of Ningbo University, Ningbo, 315010 China; 3 Department of Medical Records, The First Affiliated Hospital of Ningbo University, Ningbo, 315010 China

**Keywords:** Radical prostatectomy, Hospitalization expenses, Quantile regression, CHAID decision model, Influencing factors

## Abstract

**Objective:**

To analyze the hospitalization expenses and identify their influencing factors in patients undergoing radical prostatectomy (RP), in order to inform strategies for controlling hospitalization costs and reducing the direct economic burden on patients.

**Methods:**

A retrospective study was conducted on 4,264 inpatients who underwent radical prostatectomy (RP) in the urology department of a tertiary hospital in Zhejiang Province between January 2022 and October 2025. Basic patient demographics and detailed hospitalization cost data were extracted from hospital records. The Mann‑Whitney *U* and Kruskal‑Wallis *H* tests were performed for univariate analysis using SPSS 27.0 and StataSE 15. Subsequently, quantile regression and the CHAID decision tree model were applied to analyze hospitalization costs.

**Results:**

The total hospitalization expenses for the 4,264 RP patients were US$ 30,617,940.09, with an out-of-pocket proportion of 48.83%. Surgical costs constituted the major component. Univariate analysis identified several factors associated with expenses, including lymph node dissection (LND), blood transfusion, operative time, age, length of stay (LOS), pathological stage, choice of robotic surgery, Gleason score, Charlson Comorbidity Index (CCI), and different surgical team. Quantile regression analysis revealed that LND, blood transfusion, Barthel Index score (≤ 40), LOS (8–14 days and >14 days), robotic surgery, and surgical team (Team 2 and Team 4) had a significant impact on the three quantiles of total hospitalization cost (*Q*_*10*_, *Q*_*50*_, *Q*_*90*_) (*P* < 0.05). Both quantile regression and the Chi-squared Automatic Interaction Detection (CHAID) decision tree model identified LOS, choice of robotic surgery, and Gleason score as primary influencing factors.

**Conclusions:**

The economic burden of hospitalization for RP patients in this study was substantial, with surgical costs accounting for a large proportion and out‑of‑pocket payments representing nearly half of total expenses. LOS, robotic surgery, and Gleason score were identified as the primary drivers. These findings underscore the importance of implementing cost-containment strategies that address the key contributors to expenses, while ensuring that the quality of care is not compromised.

## Introduction

Prostate cancer ranks as the second most common cancer and the fifth leading cause of cancer-related death among men worldwide [1, 2]. It is characterized by high incidence, mortality rates and recurrence rates. Additionally, it imposes a substantial financial burden on patients and healthcare systems. In China, its incidence has been rising steadily in recent years. This trend is attributed to economic development, the adoption of a western lifestyle, and the active promotion of prostate-specific antigen (PSA) screening. According to global cancer statistics for 2022 [3, 4], the incidence of prostate cancer in China increased by 95.2% from 1990 to 2019, nearly twice the global increase (47.2%). Data from 2020 to 2022 further indicate that the age-standardized incidence rate has continued to rise [5]. Regarding prognosis, the five-year survival rate for prostate cancer patients in China is 66.4%, substantially lower than that in the United States (97.5%) [6]. Among all cancer patients in China, total hospitalization costs amounted to $2,925.60 per household, and annual medical expenditures accounted for 54.63% of disposable income, and prostate cancer imposes a severe financial burden, with annual medical expenses consuming 35%-80% of a family’s disposable income and pushing 38.7% of affected families into poverty [7–9].

Radical prostatectomy is a standard treatment for localized prostate cancer [10]. Although minimally invasive techniques, particularly robot-assisted surgery, have improved surgical precision, they have also contributed to rising hospitalization costs. For instance, one study suggests that among patients younger than 65 years, robotic surgery is associated with a 1.6-fold higher risk of postoperative financial toxicity [11]. From a health policy perspective, this trend not only strains the sustainability of insurance funds but also exposes patients and their families to substantial out-of-pocket costs.

China has established a basic health insurance system with universal coverage, consisting mainly of the Urban Employee Basic Medical Insurance, Urban Resident Basic Medical Insurance and the New Rural Cooperative Medical Scheme. A series of policy measures, including updates to the reimbursement lists, increases in reimbursement rates, and expansion of benefit packages, have been implemented to improve access to cancer care. However, in practice, out-of-pocket costs vary among patients due to differences in insurance type, coverage limits, and access to supplemental insurance (e.g., critical illness insurance or commercial health plans). For example, high-value consumables used in prostate cancer surgery, such as robotic surgery-specific supplies, staplers, and trocars, may be partially or completely excluded from the reimbursement list or may exceed payment caps, forcing patients to bear costs themselves. Thus, despite universal basic medical insurance coverage, some patients with prostate cancer may still incur high hospitalization costs.

We hypothesize that hospitalization costs for patients undergoing radical prostatectomy show substantial variation and are influenced by demographic characteristics, socioeconomic status, and clinical factors. Although prior studies have examined hospitalization costs for prostate cancer inpatients [12–14], evidence derived from Eastern China remains scarce. Guided by health economics principles, this study aims to systematically investigate the determinants of hospitalization costs based on a sample of 4,264 patients undergoing surgery from a tertiary hospital in Zhejiang Province, China. We further characterize cost distributions and identify key drivers, thereby generating evidence to inform effective cost-control strategies.

## Methods

### Data sources

Data for this study were extracted from the medical records of patients who underwent radical prostatectomy (RP) between January 1, 2022, and October 31, 2025, using the Hospital Information System (HIS) of a tertiary hospital in Zhejiang Province.

### Inclusion and exclusion criteria

Patients were identified from the HIS based on an admission diagnosis of prostate malignancy and a hospitalization period between January 1, 2022 and October 31, 2025 (*n* = 4,474). We excluded 169 patients whose primary treatment was not RP and 41 patients with incomplete data, leaving a final sample of 4,264 patients.

### Research methods

A retrospective case study method was employed. The HIS was used to obtain patients’ total hospitalization costs and detailed itemized costs, including diagnostic costs, non-surgical treatment costs, surgical treatment costs, medication costs, blood product costs, material costs, rehabilitation costs, comprehensive medical service costs, nursing costs, and other costs. Patient information was recorded in three domains: (1) Baseline data: age, occupation, marital status, insurance type, length of stay (LOS), pre-admission status, and admission route. (2) Health status: Barthel Index score, Charlson Comorbidity Index (CCI), Gleason score, and pathological stage; and (3) Surgery details: reoperation, lymph node dissection (LND), blood transfusion, ICU admission, operative time, blood loss, choice of robotic surgery and different surgical teams.

### Statistical methods

SPSS (version 27.0) and StataSE (version 15) were used for analysis. Continuous variables, particularly cost-related indicators with a skewed (non-normal) distribution, were presented as median (interquartile range, IQR) values. Categorical variables were summarized as frequencies and percentages. The Mann-Whitney *U* test was used for comparisons between two groups, and the Kruskal-Wallis *H* test was used for comparisons among multiple groups. Based on previous research, the 10th, 50th, and 90th percentiles were selected to represent low, medium, and high hospitalization costs, respectively [15, 16]. Quantile regression was employed to quantify the effects of the selected predictors at different percentiles of hospitalization costs. The Chi-squared Automatic Interaction Detection (CHAID) decision tree model was used to visually display how combinations of variables correspond to high or low hospitalization costs, providing an intuitive, tree‑based illustration that complements the regression results.

The distribution of hospitalization costs typically exhibits a marked right-skewed pattern. Therefore, policymakers are concerned not only with factors driving average costs but also with those leading to exceptionally high expenditures. Quantile regression addresses this need by estimating the differential effects of explanatory variables across various quantiles of the cost distribution, revealing heterogeneous effects often obscured in conventional mean-based analyses [17]. The CHAID algorithm was employed to identify major influencing factors. Its core operation involves recursively splitting the sample based on associations between the outcome and explanatory variables, using statistical tests (e.g., chi-squared) to automatically determine optimal groupings. The process generates an interpretable tree graph, beginning with a root node representing the entire dataset. The algorithm then creates branches (child nodes) by partitioning the data based on significant explanatory variables. When no further significant subgroups can be identified at a node, that node is considered a terminal node or leaf [18].

### Inflation adjustment

To eliminate the effects of inflation, all cost data were adjusted using the Consumer Price Index (CPI) for Zhejiang Province, as published by the National Bureau of Statistics of China for the years 2022–2025, thereby enabling more accurate cost comparisons. With the 2022 CPI set as the baseline, the CPI values for 2023, 2024 and 2025 were 100.3%, 100.3%, and 101.2%, respectively [19]. All monetary amounts are presented in US dollars (US$).

### Statement of ethics

This study was approved by the Ethics Committee of the First Affiliated Hospital of Ningbo University (approval number: 2026-R135-01). Informed consent was not required because all research data were extracted from a hospital database without critical private information about individuals for our study.

## Results

### Patient basic characteristics

The study included 4,264 patients who underwent RP, with a mean age of 70.1 years and a mean length of stay (LOS) of 8.9 days. Most patients were admitted via outpatient admission, were married, and were retired. Urban Employee Basic Medical Insurance was the predominant payment method. Pathological stage pT2 and a Gleason score of 8 were the most common findings. Perioperative events, including reoperation, blood transfusion, and ICU admission, each occurred in fewer than 2% of patients, whereas LND was performed in 14.75% of cases. Operative time was ≤ 120 min in the majority of patients (62.52%), and robot-assisted surgery was used in 75.23% of cases. The most experienced surgical team performed over half of the procedures (55.63%).

### Hospitalization expenses information

The total hospitalization cost for the 4,264 patients was US$ 30,617,940.09, of which out-of-pocket payments accounted for US$ 14,951,115.54 (48.83%). The cost per patient ranged from US$ 2,476.44 to US$ 18,090.71, with a median of US$8,268.13 (*P*_*25*_ = US$4,079.80; *P*_*75*_ = US$ 8,622.43). Surgical treatment costs constituted the largest component, followed by diagnostic and material costs (Table 1).

**Table 1:** Cost structure for patients undergoing radical prostatectomy (US$)

Component	Frequency (*n*)	Estimated cost (US$)	Percentage of total cost (%)
diagnostic costs	4,264	3,852,361.90	12.58
non-surgical treatment costs	4,264	590,780.69	1.93
surgical treatment costs	4,264	20,716,443.50	67.66
medication costs	4,264	1,177,862.34	3.85
blood product costs	179	38,178.34	0.12
material costs	4,264	3,230,958.13	10.55
rehabilitation costs	418	1,765.77	0.01
comprehensive medical service costs	4,264	604,420.19	1.97
nursing costs	4,264	274,420.92	0.90
other costs	4,256	130,748.31	0.43

*All costs are expressed in 2022 US dollars (US$)

### Univariate analysis

Univariate analysis showed that LND, blood transfusion, operative time, age, LOS, Gleason score, CCI, pathological stage, choice of robotic surgery, and surgical team were significantly associated with total hospitalization cost (all *P* < 0.05) (Table 2).

**Table 2:** Univariate analysis of hospitalization costs for patients undergoing radical prostatectomy (US$)

Variable	Frequency (*n*)	Proportion(%)	Estimated cost [M (*P*_25_, *P*_75_)]	H/Z-value	*P*-value
Reoperation (Yes/No)				-0.782	0.434
Yes	44	1.03	8267.45(4140.96,8619.81)		
No	4220	98.97	8230.01(3583.72,8894.19)		
LND (Yes/No)				-15.632	<0.001^a^
Yes	629	14.75	8205.96(3578.68,8536.63)		
No	3635	85.25	8668.76(8039.69,9607.68)		
Blood transfusion (Yes/No)				-3.995	<0.001^a^
Yes	60	1.41	8263.90(3994.24,8619.30)		
No	4204	98.59	8581.60(8245.02,9051.22)		
ICU admission (Yes/No)				-1.096	0.273
Yes	14	0.33	8267.45(4109.09,8620.86)		
No	4250	99.67	8447.57(4064.42,9655.28)		
Operative time (min)				60.166	<0.001^a^
≤ 120	2666	62.52	8237.67(6901.50,8539.80)		
121–180	1395	32.72	8299.38(3402.79,8714.55)		
>180	203	4.76	8792.86(7292.22,9410.77)		
Blood loss (ml)				6.986	0.072
≤ 50	3935	92.28	8260.68(5273.88,8607.73)		
51–200	289	6.78	8420.93(3354.07,8806.50)		
201–300	16	0.38	8472.36(4221.51,9517.33)		
>300	24	0.56	8452.84(4938.96,8915.71)		
Admission route				-0.879	0.379
Emergency	5	0.12	7064.97(3203.52,8572.19)		
Outpatient	4259	99.88	8268.80(4125.93,8622.48)		
Pre-admission status (Yes/No)				-1.322	0.186
Yes	2009	47.12	8262.76(3836.52,8595.52)		
No	2255	52.88	8281.30(4597.77,8645.76)		
Pathological stage				-18.925	<0.001^a^
pT2	3736	87.62	8323.46(7152.91,8648.91)		
pT3	528	12.38	3212.43(3034.77,3630.88)		
Robotic surgery (Yes/No)				-47.282	<0.001^a^
Yes	3208	75.23	3138.10(3006.10,3334.86)		
No	1056	24.77	8430.86(8108.07,8792.86)		
Age(yr)				12.410	0.006^a^
≤ 59	221	5.18	8368.01(7175.34,8756.36)		
60–69	1647	38.63	8241.95(6920.10,8590.20)		
70–79	2193	51.43	8291.50(3549.60,8628.13)		
>79	203	4.76	8262.77(3579.69,8782.00)		
Marital status				3.039	0.386
Married	4171	97.82	8272.63(4125.93,8622.48)		
Divorced	15	0.35	8463.30(3469.53,9072.11)		
Widowed	60	1.41	8164.61(4203.91,8680.57)		
Other	18	0.42	7707.28(3247.46,8303.75)		
Length of stay (days)				231.099	<0.001^a^
1–7	1568	36.77	8108.03(5678.32,8425.01)		
8–14	2455	57.58	8347.11(3621.41,8711.40)		
>14	241	5.65	8997.13(7827.97,9833.07)		
Occupation				1.528	0.466
Retired	2638	61.87	8299.81(4203.91,8624.96)		
Farmer	48	1.13	8334.37(4246.27,8644.10)		
Other	1578	37.00	8212.20(3758.24,8618.48)		
Insurance type				1.404	0.496
Urban employee medical insurance	2666	62.52	8292.20(5580.91,8648.91)		
Urban resident medical insurance	1572	36.87	8249.50(3629.07,8591.19)		
Self-financed	26	0.61	8382.03(6376.55,8613.57)		
Barthel Index score				0.140	0.932
>60	3956	92.78	8272.36(4131.58,8620.10)		
41–60	304	7.13	8262.61(3430.11,8651.05)		
≤ 40	4	0.09	8221.55(8212.57,8230.52)		
Gleason score				2056.311	<0.001^a^
≤ 6	80	1.88	2921.04(2691.44,8431.49)		
3་4	149	3.49	3092.32(2983.03,3209.40)		
4་3	796	18.67	3138.44(3009.62,3321.87)		
8	3077	72.16	8395.52(7977.97,8728.47)		
9–10	162	3.80	8808.64(8601.66,9098.76)		
CCI				182.334	<0.001^a^
≤ 1	2152	50.47	8210.13(3463.59,8554.32)		
2–3	1778	41.70	8236.85(3626.70,8620.43)		
>3	334	7.83	8615.62(8380.22,9092.47)		
Surgical team				777.251	<0.001^a^
Team 1	2372	55.63	8425.73(8007.91,8804.34)		
Team 2	518	12.15	3320.88(3072.27,7560.13)		
Team 3	792	18.57	8133.98(3405.59,8577.25)		
Team 4	582	13.65	4598.96(3102.27,8273.69)		

*All costs are expressed in 2022 US dollars (US$). ^a^*P* < 0.05

Abbreviations: *LND* Lymph node dissection; ICU Intensive care unit; *CCI* Charlson Comorbidity Index

### Quantile regression analysis

LND, blood transfusion, operative time, age, LOS, pathological stage, choice of robotic surgery, Gleason score, CCI, and surgical team were included as independent variables based on the univariate analysis results. In addition, based on clinical importance and a review of the literature [20–25], six clinically relevant variables—Barthel Index score, insurance type, occupation, marital status, ICU admission, and reoperation—were incorporated into the model regardless of statistical significance.

LND, blood transfusion, Barthel Index score (≤ 40), LOS (8–14 days and >14 days), robotic surgery, and surgical team (Team 2 and Team 4) were significantly associated with hospitalization costs across all three quantiles (*Q*_10_, *Q*_50_, *Q*_90_) (*P* < 0.05). Specifically, LND was associated with increased costs at all quantiles, with a larger effect at the higher quantile (*Q*_90_: 0.077) than at the lower quantile (*Q*_10_: 0.030). Blood transfusion also increased costs, with the greatest effect observed at *Q*_10_ (0.142). A Barthel Index score ≤ 40 significantly increased costs at *Q*_10_ (0.130) but decreased costs at Q50 and Q90 (-0.024 and − 0.111, respectively). For LOS >14 days, the coefficient increased progressively across quantiles. Robotic surgery was associated with substantially higher costs at all quantiles (coefficients ranging from 0.715 to 0.831). All associations were statistically significant (Table 3).

**Table 3:** Quantile regression analysis of hospitalization costs for patients undergoing radical prostatectomy (US$)

Variable	Hospitalization costs		
	Q_10_	Q_50_	Q_90_
Reoperation (Yes/No) (contrast: No)			
Yes	-0.029	0.032^a^	0.023
LND (Yes/No) (contrast: No)			
Yes	0.030^a^	0.020^a^	0.077^a^
Blood transfusion (Yes/No) (contrast: No)			
Yes	0.142^a^	0.046^a^	0.112^a^
ICU admission (Yes/No) (contrast: No)			
Yes	0.107^a^	0.043^a^	-0.032
Operative time (contrast: ≤120)			
121–180	0.009	0.013^a^	0.006
>180	0.012	0.030^a^	0.070^a^
Pathological stage (contrast: pT2)			
pT3	0.029	0.011^a^	0.021
Age(contrast: ≤59)			
60–69	-0.019	-0.008^a^	-0.080^a^
70–79	-0.011	0.001	-0.080^a^
>79	-0.009	0.015^a^	-0.061^a^
Marital status(contrast: Married)			
Divorced	0.019	0.023	-0.092^a^
Widowed	-0.025	0.006	-0.011
Other	-0.076	-0.100^a^	-0.042
Occupation(contrast: Retired)			
Farmer	-0.015	0.003	0.053
Other	-0.006	0.001	0.013
Insurance type(contrast: Urban employee medical insurance)			
Urban resident medical insurance	0.008	0.001	0.007
Self-financed	0.015	-0.003	-0.016
Barthel Index score(>60)			
41–60	-0.004	0.012^a^	-0.039
≤ 40	0.130^a^	-0.024^a^	-0.111^a^
Length of stay (contrast: ≤7)			
8–14	0.032^a^	0.026^a^	0.076^a^
>14	0.086^a^	0.105^a^	0.251^a^
Gleason score(contrast: ≤6)			
3་4	0.079^a^	0.085	-0.277^a^
4་3	0.040	0.089	-0.234^a^
8	0.247^a^	0.249^a^	0.027
9–10	0.365	0.258^a^	-0.073^a^
Robotic surgery (Yes/No) (contrast: No)			
Yes	0.750^a^	0.831^a^	0.715^a^
CCI (contrast: ≤1)			
2–3	0.003	0.004	0.008
>3	0.001	0.007^a^	0.045
Surgical team (contrast: Team 1)			
Team 2	-0.023^a^	0.012^a^	-0.051^a^
Team 3	-0.002	-0.003	-0.041^a^
Team 4	-0.029^a^	-0.035^a^	-0.079^a^

*All costs were expressed in 2022 US dollars (US$). ^a^*P* < 0.05

Abbreviations: *LND* Lymph node dissection; ICU Intensive care unit; *CCI* Charlson Comorbidity Index

### Development of the CHAID decision tree model

The variables included in the quantile regression model were also used to construct a CHAID decision tree model to identify homogeneous subgroups of hospitalization costs. Pre-pruning parameters were set as follows: a minimum of 200 cases for parent nodes and 100 for child nodes, a maximum tree depth of three levels, and a significance level of 0.05 for node splitting [26, 27]. Model performance was evaluated using 10-fold cross-validation. The final model retained three variables: LOS, choice of robotic surgery, and Gleason score.

The decision tree identified distinct patient subgroups with significantly different cost profiles (*P* < 0.05 for all inter-group comparisons). Patients who underwent robotic surgery, had a Gleason score ≥ 8, and a LOS > 7 days incurred the highest costs. In contrast, the lowest costs were observed among patients who did not undergo robotic surgery and had a Gleason score ≤ 6 or 3 + 4.

**Fig. 1:**
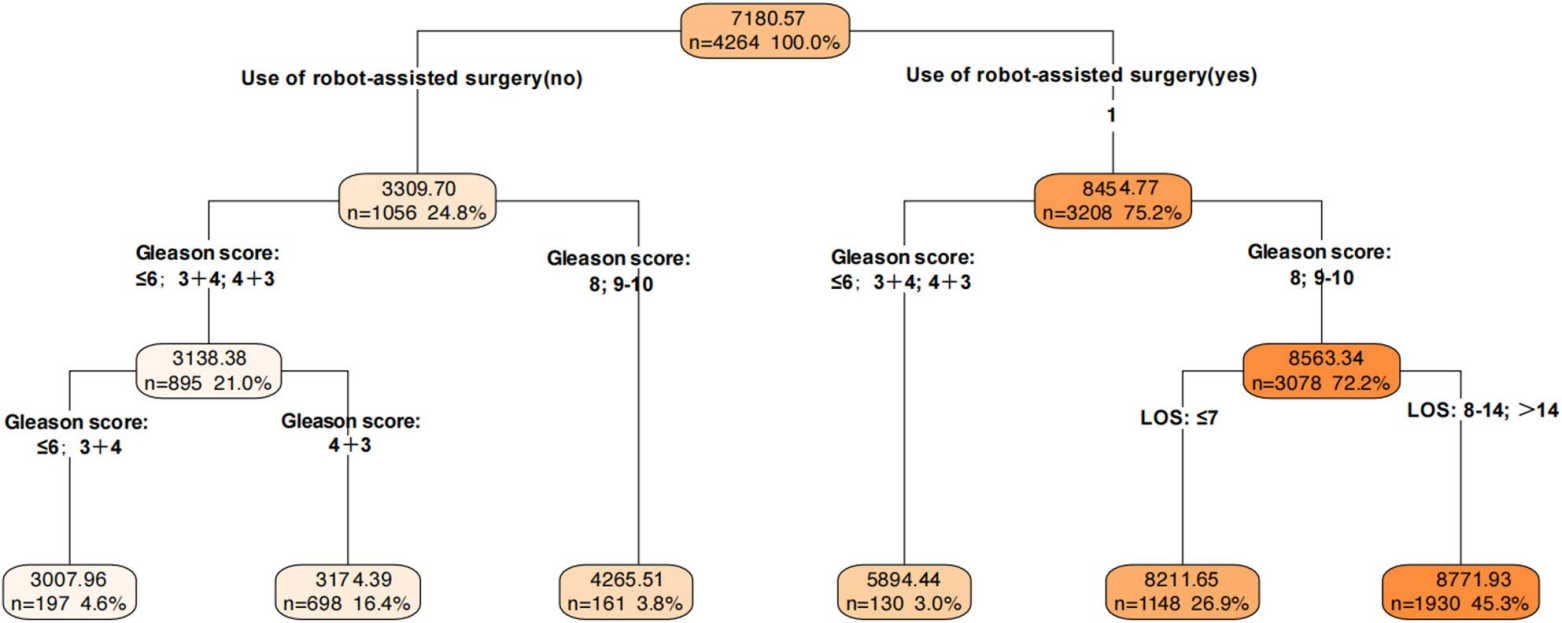
CHAID decision tree model for hospitalization costs in patients undergoing radical prostatectomy. (*All costs are expressed in 2022 US dollars (US$). LOS: Length of stay.)

After 10-fold cross-validation, the model showed a relative error of 0.186 in the training set and 0.187 in the cross-validation set (SD = 0.014). The training error and cross‑validation error were highly comparable (difference < 0.002), indicating no evidence of overfitting and suggesting satisfactory generalizability. With a goodness‑of‑fit level (*R²* = 0.814) on the training set, this model is suitable for predicting medical costs (Fig. 1).

## Discussion

Our analysis of 4,264 RP cases confirms the significant economic burden associated with this treatment, with surgical, diagnostic, and material costs constituting the primary expenditures. A study conducted in Liaoning province, China, also found a similar composition of hospital costs for cancer patients [28]. We employed quantile regression to examine how the effects of predictors vary across the cost distribution, complemented by a CHAID decision tree that visually identifies combinations of variables associated with cost subgroups. This combined approach offers a more nuanced understanding than traditional linear regression, which only captures mean effects. We identified LOS, Gleason score, and the adoption of robotic-assisted surgery as three core factors independently associated with total hospitalization costs. These findings provide critical insights into current patterns of resource utilization in prostate cancer surgery and inform strategies for optimizing clinical pathways and cost management.

Regarding population characteristics, the patients undergoing RP in this study were predominantly elderly, married, and covered by basic medical insurance. The most common cancer stage was pT2, and the predominant Gleason score was 8. Prior research suggests that RP offers survival benefits for patients with localized prostate cancer and a life expectancy exceeding 10 years, whereas those with high-risk or locally advanced disease are more likely to benefit from surgery. Elderly patients often present with more advanced tumor stages and higher grades compared to younger patients, making RP a more common choice for high-risk localized prostate cancer [29]. The low proportion of pT3 stage (12.38%) and the limited use of LND (14.75%) suggest that most patients in this cohort had localized disease and were assessed preoperatively to have a low risk of lymph node metastasis. This aligns with current clinical guidelines [30, 31]. This indicates that the patient selection for surgery in this study largely adhered to clinical standards for treating localized prostate cancer. Regarding surgical approach, a substantial majority of patients (75.23%) opted for robotic surgery, reflecting the widespread adoption of this minimally invasive technology in major medical centers across China [32, 33]. This trend not only highlights technological advancement but also serves as a significant structural factor influencing medical costs.

In terms of cost-influencing factors, the quantile regression analysis provided deeper insights than traditional linear regression. It showed that LOS > 14 days progressively increased costs across quantiles, with the largest coefficient observed at the 90th percentile (0.251). This suggests that the extended LOS for these patients may be associated with more complex conditions or complications. As a large tertiary hospital, the management of complex cases often necessitates longer hospital stays, which in turn drives up cumulative costs such as those for medications and daily bed charges. In our cohort, the mean LOS was 8.9 days (with 47.12% of patients undergoing pre‑admission), which is substantially longer than the typical postoperative hospital stay in both American and European healthcare systems, where robotic prostatectomy patients are often discharged after one night or even on the same day of surgery [34]. This marked difference in care models significantly limits the generalizability of our findings to Western healthcare systems. Nevertheless, consistent with our results, a study from the United States has demonstrated that prolonged length of stay is associated with higher costs of disposable supplies and longer operative time [35]. Therefore, reducing LOS without compromising care quality may be a critical strategy for cost control.

For robotic surgery, the cost‑increasing effect was substantial across all quantiles, but its impact was most pronounced when combined with longer LOS and higher Gleason score, as demonstrated by the CHAID decision tree. This suggests that robotic surgery alone does not independently drive costs, and the consistently higher costs associated with robotic surgery likely reflect the fixed costs of the robotic platform (e.g., equipment depreciation, disposable instruments) rather than excessive clinical resource utilization. From a health policy perspective, the incremental cost of robotic surgery should be weighed against its potential benefits [36]. Studies have consistently demonstrated that robot-assisted RAP reduces LOS compared to open surgery [37, 38]. In fact, same-day discharge following robotic radical prostatectomy has emerged as a safe and feasible option, successfully implemented in various countries, in Europe and North America [39, 40]. However, in China, same-day discharge for robot-assisted RAP remains in the exploratory stage. This disparity can largely be attributed to several structural factors within the domestic healthcare system: (1) reimbursement policies that favor inpatient care over outpatient services; (2) cultural preferences for hospital‑based recovery, with concerns about managing catheters at home; and (3) limited availability of community‑based or home‑based postoperative support. To maximize the cost‑saving potential of robotic surgery without compromising quality, policy interventions should aim to address these systemic barriers. Examples include redesigning care pathways to transition appropriate patients to outpatient settings, adjusting insurance coverage to promote outpatient recovery, and developing home catheter management programs.

LND was associated with increased costs at all three quantiles, with the effect most pronounced in the high‑cost group. A plausible explanation is that patients in the high‑cost group are more likely to have high‑risk or locally advanced prostate cancer, for whom more extensive LND (e.g., extended pelvic LND) is typically performed. More extensive LND requires longer operative time, more disposable supplies, and may increase the risk of postoperative complications, all of which contribute to higher costs. Conversely, patients in the low‑cost group who undergo LND may only receive limited sampling, which adds less to the overall expense.

Blood transfusion increased costs across all quantiles, with the highest coefficient observed at *Q*_*10*_ (0.142). While a single transfusion could substantially increase expenses for patients in the low‑cost group, the small sample size (*n* = 60, 1.41%) limits definitive conclusions. Similarly, the Barthel Index score ≤ 40 group (*n* = 4, 0.09%) showed an unstable pattern: it increased costs at the low quantile (0.130) but decreased costs at the middle and high quantiles (− 0.024 and − 0.111, respectively). Given the very limited sample sizes in both subgroups, these coefficients should be interpreted cautiously and validated in larger studies.

Notably, this study also examined the potential influence of surgical team proficiency on patient costs. The quantile regression analysis revealed significant cost differences between surgical teams (Team 2 and Team 4) across all quantiles, indicating that team-specific practice patterns affect costs. However, the surgical team variable is a composite indicator that may reflect multiple underlying factors. The observed cost differences could reflect variations in case complexity (e.g., more complex or high-risk patients may be preferentially assigned to senior teams), surgeon experience and learning curve (e.g., more experienced teams may operate more efficiently or use resources differently). Interestingly, a study by Sorber et al. [41] demonstrated that cost awareness of common surgical supplies is severely impaired among all members of the surgical team, suggesting that even experienced teams may lack accurate cost information, which could contribute to unwarranted practice variation. Differential utilization of robotic surgery (although robotic surgery was included as a separate covariate, its use may still vary across teams), and perioperative management protocols (e.g., transfusion or discharge) may also contribute. Due to the retrospective design, we could not fully disentangle these components. Therefore, the surgical team variable should be interpreted as a global measure of practice pattern variation rather than a direct indicator of any single factor. Future research linking detailed intraoperative metrics and surgeon‑specific characteristics is needed to better understand the drivers of inter‑team cost differences. Nonetheless, standardizing surgical procedures and enhancing team training could reduce unwarranted cost variation and improve healthcare value [42].

Based on the decision tree model, this study further visualized the interaction of multiple factors, identifying typical patient subgroups with the highest and lowest hospitalization costs. The highest-cost subgroup (robotic surgery, Gleason score ≥ 8, and LOS > 7 days) suggests that when robotic surgery is combined with prolonged LOS and a high Gleason score, total costs are significantly increased. The lowest-cost subgroup (non-robotic surgery and Gleason score ≤ 6 or 3 + 4) represents patients with relatively low-risk disease, conventional surgical plans, and smooth recovery. Compared to the quantile regression results, the decision tree model selected only three splitting variables (LOS, robotic surgery, and Gleason score); all other candidate variables were excluded. This indicates that within the cohort, the discriminatory power of these unselected variables was limited after accounting for the three dominant factors. The quantile regression model prespecified all clinically relevant variables as independent covariates and estimated their net effects at different cost quantiles after controlling for other factors. In contrast, the decision tree model aims to identify the variable and split point that best improve the sample segmentation purity. Thus, after initial segmentation by “choice of robotic surgery,” the additional discriminatory ability of the other variables became insufficient to warrant further splitting. This demonstrates that integrating quantile regression with a CHAID decision tree model can uncover the most dominant factors influencing hospitalization costs from complementary perspectives.

This study has several limitations. First, the data were drawn from a large tertiary hospital, where the proportion of high‑technology applications and complex cases is likely higher than the general average. This may limit the generalizability of the findings to broader settings. Second, the analysis measured only direct hospitalization costs and did not capture subsequent expenses related to postoperative complication management or long‑term follow‑up. Consequently, the actual total treatment cost for RP is likely higher than reported here. Third, despite incorporating clinically important variables based on literature review, residual confounding from unmeasured factors, such as detailed surgeon experience, cannot be entirely excluded due to the retrospective design. Fourth, due to data limitations, we were only able to obtain total out-of-pocket payments, rather than a detailed breakdown of which specific items were uncovered. Future studies linking granular insurance claims data or patient billing records are necessary to clarify the specific components of the financial burden on patients.

## Conclusion

This study analyzed hospitalization expenses for 4,264 patients undergoing radical prostatectomy at a tertiary hospital in Eastern China from 2022 to 2025, examining the cost structure and influencing factors. Our findings, which integrated quantile regression with the CHAID decision tree model, confirm the hypothesis that hospitalization costs for RP are influenced by demographic, socioeconomic, and clinical factors, with LOS, robotic surgery, and Gleason score as dominant drivers. Notably, out‑of‑pocket payments accounted for nearly half (48.83%) of total expenses, underscoring the substantial financial burden on patients. These findings contribute to a deeper understanding of the determinants of inpatient costs for RP, informing hospital resource allocation, clinical pathway optimization, and insurance reimbursement strategies. Identifying patients at risk of high expenditures could also guide future research into cost‑reduction strategies, such as standardized perioperative protocols and enhanced recovery programs. Further integration of economic data with clinical decision‑making and health policy is essential for developing evidence‑based strategies that contain costs without compromising care quality.

## Data Availability

Datasets used or analyzed during the current study are available from the corresponding author on reasonable request.

## Abbreviations

RP: Radical Prostatectomy
RT: Radiation Therapy
ADT: Androgen Deprivation Therapy
HIS: Hospital Information System
PSA: Prostate-specific Antigen
CHAID: Chi-squared Automatic Interaction Detection
CPI: Consumer Price Index
IQR: Interquartile Range
LOS: Length of stay
CCI: Charlson Comorbidity Index
LND: Lymph node dissection
